# Combined Use of Guanfacine and *N*-Acetylcysteine for the Treatment of Cognitive Deficits After Traumatic Brain Injury

**DOI:** 10.1089/neur.2023.0124

**Published:** 2024-03-13

**Authors:** Siddharth Khasnavis, Timothy Belliveau, Amy Arnsten, Arman Fesharaki-Zadeh

**Affiliations:** ^1^ Department of Psychiatry, Yale School of Medicine, New Haven, Connecticut, USA.; ^2^ Department of Neurology, Yale School of Medicine, New Haven, Connecticut, USA.

**Keywords:** cognitive symptoms, guanfacine, *N*-acetylcysteine, traumatic brain injury

## Abstract

Traumatic Brain Injury (TBI) is a significant contributor to disability across the world. TBIs vary in severity, and most cases are designated mild TBI (mTBI), involving only brief loss of consciousness and no intracranial findings on imaging. Despite this categorization, many persons continue to report persistent cognitive changes in the months to years after injury, with particular impairment in the cognitive and executive functions of the pre-frontal cortex. For these persons, there are no currently approved medications, and treatment is limited to symptom management and cognitive or behavioral therapy. The current case studies explored the use of the alpha-2A adrenoreceptor agonist, guanfacine, combined with the antioxidant, *N*-acetylcysteine (NAC), in the treatment of post-TBI cognitive symptoms, based on guanfacine's ability to strengthen pre-frontal cortical function, and the open-label use of NAC in treating TBI. Two persons from our TBI clinic were treated with this combined regimen, with neuropsychological testing performed pre- and post-treatment. Guanfacine + NAC improved attention, processing speed, memory, and executive functioning with minimal side effects in both persons. These results encourage future placebo-controlled trials to more firmly establish the efficacy of guanfacine and NAC for the treatment of cognitive deficits caused by TBI.

## Introduction

Traumatic brain injury (TBI) is a major cause of mortality and disability worldwide.^1^ In the United States alone, the Centers for Disease Control and Prevention estimated 2.5 million emergency department visits related to TBI in 2010.^2^ A growing body of literature reveals long-term cognitive and functional impacts from TBI, extending to a higher risk of neurodegenerative disease.^3^ Such risk may be amplified through repetitive injury.^4^

TBIs encompass a host of neurological insults. Severe TBIs can be accompanied by intracerebral contusions or hemorrhage and axonal disruption within the brain, described as diffuse axonal injury.^5^ In most cases, categorized as mild TBI (mTBI), there can be disruption of neural circuits involved in behavioral and cognitive functions.^6^ The resulting cognitive impairment can involve the broader domain or subcomponents of executive function that depend on the pre-frontal cortex (PFC).^7^ Whereas most persons improve in weeks to months post-injury, up to 15% may report persistent symptoms beyond a year.^8^ Behavioral symptoms also accompany cognitive changes and include apathy, irritability, impulsivity, and depression.^4,9^

Advances in imaging reveal alterations in brain structure and connectivity post-TBI. Executive function deficits associate with microstructural changes to white matter tracts connecting the dorsolateral pre-frontal cortex (DLPFC) to other regions of the brain.^10^ Diminished activation of the left DLPFC is particularly associated with poor planning after TBI.^11^ This association between the DLPFC and executive function is shared with other conditions and observed in normal aging.^12^ Rodent models illustrate this particularly well, revealing excessive cyclic adenosine monophosphate/protein kinase A (cAMP-PKA) signaling and loss of dendritic spines in the PFC after posterior cortical traumatic injury. This further associates with impaired working memory and emotional regulation.^13,14^ The PFC is similarly vulnerable to a loss of dendritic spines and connectivity in response to chronic stress.^15^

Amid these findings, pharmacological treatments for enhancing cognitive function after TBI are limited. One candidate of interest is the alpha-2A adrenoceptor agonist, guanfacine, marketed as Tenex (immediate release) and Intuniv (extended release) for treatment of attention-deficit hyperactivity disorder (ADHD). Animal studies show that guanfacine regulates cAMP-PKA-K^+^ signaling at post-synaptic alpha-2A receptors to enhance PFC neuronal firing, protecting PFC dendritic spines from the detrimental effects of stress.^16^ Administration of guanfacine is associated with improved working memory across species and improved impulse control and cognitive flexibility in primates.^16^ To date, only one study examined guanfacine in the treatment of cognitive symptoms after TBI. McAllister and colleagues demonstrated that in persons with mTBI versus in healthy controls, guanfacine increased activity within the right PFC during a working memory task.^17^ These persons were not followed longitudinally, and protracted benefits of treatment remain to be understood.

Stimulants have also drawn interest in the treatment of post-TBI cognitive deficits, with the combined use of methylphenidate and cognitive therapy beneficial for working memory function, attention, and executive function in a randomized controlled trial.^18^ However, this study did not explore the impact of methylphenidate alone. The routine use of stimulants may also be limited in certain practice settings or among vulnerable persons, whereas guanfacine could be used more widely.

*N*-acetylcysteine (NAC) may complement the role of guanfacine in a post-TBI treatment protocol. Intracellularly, NAC increases glutathione levels and reduces calcium overload, exerting a net antioxidant effect to protect mitochondrial function.^19^ NAC inhibits the enzymatic pathway, which leads to production of kynurenic acid (KYNA), an N-methyl-D-aspartate receptor (NMDAR) antagonist, and DLPFC neurons are especially dependent on NMDAR neurotransmission.^19,20^ Post-mortem studies show higher concentrations of KYNA in the PFC of patients with schizophrenia compared to controls, in association with greater exposure to proinflammatory cytokines.^21^ There is interest in the anti-inflammatory properties of NAC to aid in the brain's recovery from TBI.^22^ Limited trials include a randomized control trial among actively deployed soldiers with mTBI after blast exposure.^23^ In this study, NAC had a significant impact on post-injury symptoms (including neurocognitive dysfunction), especially when administered within 24 h of the insult. We propose that NAC may have a longitudinal anti-inflammatory and -oxidant effect within a post-TBI treatment protocol. Such effects are also relevant given that TBI is linked to chronic neuroinflammation in a subset of patients.^24^ Further, inhibiting KYNA production could alleviate NMDAR antagonism and improve neurotransmission through the DLPFC.^25^

Amid these neurobiological findings, there is no approved pharmacotherapy to treat cognitive symptoms after TBI.^26^ We sought to bolster literature on the treatment of chronic post-TBI symptoms and describe the combined use of guanfacine and NAC. We identified 33 persons in our practice who were recommended this protocol and who continued to follow up in our TBI clinic. At the last encounter, 12 persons continued both medications. Meanwhile, 6 remained on guanfacine alone, 11 on NAC alone, and 4 discontinued both. Among those on both, 7 reported benefits such as improved focus, working memory, and feeling less overstimulated. Three patients on guanfacine monotherapy reported improved concentration. We identified 2 persons who completed neuropsychological testing both before treatment and additionally between 3 and 20 months after starting care. Their course of recovery is summarized.

## Case 1

H.T., a 52-year-old male retired emergency responder, presented 17 months after a motor vehicle accident. During a rear-end collision, he experienced retroflexion of the neck and a forceful strike into the headrest. There was no loss of consciousness or post-traumatic amnesia and no neuroimaging gathered immediately after the accident. H.T. observed diminished focus in tasks, reduced working memory and short-term recall, as well as word-finding difficulties in the months post-injury. These changes even prompted retirement from work. He noted diminished frustration tolerance and poor sleep over time. H.T. had a past medical history of essential hypertension and lumbosacral (L5–S1) fusion by the anterior approach.

Examination revealed a normal mental status, no focal neurological findings, and a score of 22/30 on the Montreal Cognitive Assessment (MoCA).^27^ Impairments were noted on Trails B, copying of a cube, complex sentence repetition, fluency (seven words given), and reverse digit span and recall (three of five words). Neuropsychological testing was completed within 1 week of intake, and corroborated deficits in attention, concentration, and information encoding were attributed to a post-concussive syndrome and complicated by chronic pain. A magnetic resonance imaging (MRI) revealed no structural abnormalities. An overall impression of post-injury dysexecutive phenomenon led to recommendation of guanfacine extended-release (ER) 1 mg nightly and NAC 600 mg daily.

In follow-up visits at 4-month intervals, H.T. reported improvements in attention and working memory. He observed greater interest and capacity for social engagement. Given this response, the guanfacine dose was incrementally titrated to 4 mg, with NAC increased to 1200 mg, both without reported side effects. Neuropsychological examination was repeated at 20 months after initial study and showed mild improvements in attention, verbal memory, and executive functioning as outlined in Table 1.

**Table 1.** Neuropsychological Evaluation Scores for H.T. (Case 1) and S.D. (Case 2)

	***Case 1 Pre-treatment Evaluation 1***	***Case 1 Post-treatment Evaluation 2***	***Case 2 Pre-treatment Evaluation 1***	***Case 2 Post-treatment Evaluation 2***
NIHTB-CB Fluid Cognition Index (T Score)	Not administered	38	40	54^*^
Wechsler Adult Intelligence Scale-IV Working Memory Index (Standard Score)	80	89	108	114
Wechsler Adult Intelligence Scale-IV Processing Speed Index (Standard Score)	89	89	102	111
Wechsler Memory Scale-IV Logical Memory I (Scaled Score)	08	09	04	07
Wechsler Memory Scale-IV Logical Memory II (Scaled Score)	08	09	03	09
Hopkins Verbal Learning Test–Revised Total Recall (T Score)	28	46	11	22
Hopkins Verbal Learning Test–Revised Delayed Recall (T Score)	27	44	06	09
Brief Visuospatial Memory Test–Revised Total Recall (T Score)	29	29	12	21
Brief Visuospatial Memory Test–Revised Delayed Recall (T Score)	35	35	05	08
Phonemic Fluency–FAS (T Score)	42	50	25	30
Semantic Fluency–Animals (T Score)	45	63		
Trail Making Test Part A (T Score)	35	35	46	64
Trail Making Test Part B (T Score)	46	46	56	70

Changes assessed for statistical significance per Reliable Change Index and regression method outlined by Maassen and colleagues^28^ and Crawford and colleagues.^29^ Asterisk (“^*^”) represents a statistically significant change). Larger values signify better cognitive performance.

NIHTB-CB, National Institutes of Health Toolbox–Cognitive Battery.

## Case 2

S.D., a 59-year-old previously healthy male, reported concerns regarding short-term memory and multi-tasking difficulty 2 months after an assault. He was amnestic for the event, which involved multiple facial fractures, a loss of consciousness of ∼30 min, and fragmentary recollection of events that occurred during the 10 days post-injury. No intracranial abnormalities were identified on imaging. Both S.D. and his spouse acknowledged behavioral changes, including decreased empathy, increased irritability, and hypervigilance, after the event.

In our initial assessment, S.D. scored 25/30 on the MoCA with primary deficits on tests of recall, fluency, and serial calculations. Neurological exam was normal. MRI completed just before intake showed few scattered T2/fluid-attenuated inversion recovery hyperintensities, but an otherwise normal brain structure. S.D. was recommended speech and cognitive therapy, mirtazapine 7.5 mg nightly for mood and sleep, and the combination of NAC 600 mg daily and guanfacine 2 mg nightly for cognitive symptoms. A neuropsychological exam completed 4 months after his assault revealed mild slowing in executive function–based tasks and additional impairment in short-term recall.

Four months after his intake, S.D. noted improvement in mood and function at home. Neuropsychological testing repeated 9 months post-injury showed improvement in attention, processing speed, working memory, and executive function, changes that were measurable on the National Institutes of Health Toolbox–Cognitive Battery (NIHTB-CB) Fluid Cognition composite score (pre-treatment total score 40 vs. post-treatment total 54; effect size = 1.7, T value for discrepancy from predicted score of 45.0 being 1.67, *p* < 0.05). Additional scores are noted in Table 1.

S.D. reported no side effects and noted continued improvement at 11 months after his injury, eventually rejoining his previous employment. He noted less irritability and greater capacity to structure tasks. Guanfacine and NAC were continued without need for dose adjustment.

## Discussion

The cases outlined here are among a growing cohort of persons in our practice treated with guanfacine and NAC for executive dysfunction resulting after TBI. Our experience corroborates findings that a subset of patients with TBI have persistent cognitive changes beyond the acute and subacute phase of the condition. Symptoms can be difficult for persons and their treaters to manage in the absence of formally approved pharmacotherapy. We advocate for a trial of guanfacine and NAC considering their well-studied neurobiological foundation and guanfacine's proven benefit in related conditions where attention and executive function are affected. We observed that the domains with greatest improvement post-treatment included attention, executive function, and processing speed, respectively.

Guanfacine is not without adverse effects, and both our experience and the literature outline certain challenges experienced by patients. Somnolence, sedation, and fatigue are the most common adverse effects, which lead to discontinuation of the ER formulation.^30^ For instance, among Japanese adults treated with ER guanfacine for ADHD, 83.8% noted an adverse effect, though the majority were mild to moderate in severity (somnolence and increased thirst the most common).^31^ Given the higher frequency of adverse effects from the immediate-release formulation attributable to rapid absorption, the ER formulation is typically preferred.^30^ One alternative strategy is the use of the immediate-release formulation in a lower starting dose such as 0.5 mg at bedtime.^32^

NAC has been well tolerated both in our clinic and in human trials. Gastrointestinal discomfort can be the most common issue reported by persons, although such adverse effects have not differentiated from placebo in controlled studies. This was notably demonstrated in a review of a high dose of NAC used in the treatment of respiratory diseases by Calverley and colleagues.^33^

Our report is not without limitations, many intrinsic to the format of the case series. We cannot fully eliminate the impact of psychiatric conditions in the cases detailed here. This aligns with a challenge encountered in the real world. Delayed surveys of persons with mTBI reveal elevated rates of psychiatric conditions, notably anxiety, depression, and post-traumatic stress disorder, which may also be related to synapse loss in the DLPFC and associated with emotional dysregulation.^34,35^ Nonetheless, in neuropsychological testing for both our cases, TBI was identified as the primary contributor to cognitive symptoms. It is similarly not possible to exclude the effect of time and natural improvement in cognitive symptoms post-injury. Last, the cases described here include only mild- and moderate-severity TBI and treatment applied months after injury. There may be value for this pharmacotherapy in persons with severe TBI or from earlier initiation of the regimen, though still outside the acute phase of injury. These hypotheses, and the limitations outlined above, stress the need for a larger sample size and more systematic review of patients treated with guanfacine and NAC for post-TBI cognitive symptoms. Still, our report offers experiential support for the use of guanfacine and NAC in patients with TBI.

Our work is bolstered by similar experiences treating persons with post-COVID cognitive dysfunction. Emerging studies reveal executive dysfunction to be a key element of cognitive change after COVID infection.^36^ We have described 12 persons prescribed guanfacine and NAC for chronic post-COVID symptoms, many reporting similar benefits in executive function, working memory, and multi-tasking capability.^37^ The combination of neuroinflammation and PFC dysfunction may be a shared element between TBI and post-COVID cognitive dysfunction, offering a broader utility for the pharmacological combination described here.^38^

## Conclusion

There is a pressing need for pharmacotherapy that can treat persistent cognitive symptoms post-TBI. We propose that guanfacine and NAC can be used in combination to improve pre-frontal cortical function and attenuate the inflammatory response after TBI. Two cases from our cohort of persons treated with this regimen highlight improvement in routine function and measurable change on neuropsychological testing.

## Transparency, Rigor, and Reproducibility Summary

This case series was derived through a retrospective review of persons who presented to a single clinician with cognitive concerns after traumatic brain injury and were additionally recommended the described therapeutic regimen. In light of this format, neither the study nor an analytical plan were pre-registered.^1,2^ Additionally, the study was not powered to demonstrate a statistically significant outcome and no blinding was utilized.^3–5^ Individuals suitable for discussion in this case series were identified by surveying the clinician's panel without any restriction of a time period. This inclusion and any clinical outcomes were assessed by clinicians with expertise in the evaluation and treatment of TBI. Within the two described cases, pre- and post-treatment neuropsychological outcomes were examined using the Reliable Change Index and regression method.^6^ Statistical analysis pertinent to neuropsychological evaluation were conducted by a study co-author with expertise in the subject matter.^9^ We do not anticipate any barriers in the reproducibility of our approach in the appropriate clinical setting.^7^ Neither external validation nor data and code availability were applicable for this study format.^11–13^

## Abbreviations Used

ADHD: attention-deficit hyperactivity disorder
cAMP: cyclic adenosine monophosphate
DLPFC: dorsolateral pre-frontal cortex
ER: extended-release
KYNA: kynurenic acid
MoCA: Montreal Cognitive Assessment
MRI: magnetic resonance imaging
mTBI: mild TBI
NAC: *N*-acetylcysteine
NIHTB-CB: National Institutes of Health Toolbox–Cognitive Battery
NMDAR: N-methyl-D-aspartate receptor
PFC: pre-frontal cortex
PKA: protein kinase A
TBI: traumatic brain injury
